# Host SUMOylation Pathway Negatively Regulates Protective Immune Responses and Promotes *Leishmania donovani* Survival

**DOI:** 10.3389/fcimb.2022.878136

**Published:** 2022-06-06

**Authors:** Jhalak Singhal, Evanka Madan, Ayushi Chaurasiya, Pallavi Srivastava, Niharika Singh, Shikha Kaushik, Amandeep Kaur Kahlon, Mukesh Kumar Maurya, Manisha Marothia, Prerna Joshi, Anand Ranganathan, Shailja Singh

**Affiliations:** Special Centre for Molecular Medicine, Jawaharlal Nehru University, New Delhi, India

**Keywords:** autophagy, SUMOylation, host–pathogen interaction, autophagy maturation, *Leishmania donavani*, SUMOylation mediated immune responses

## Abstract

SUMOylation is one of the post-translational modifications that have recently been described as a key regulator of various cellular, nuclear, metabolic, and immunological processes. The process of SUMOylation involves the modification of one or more lysine residues of target proteins by conjugation of a ubiquitin-like, small polypeptide known as SUMO for their degradation, stability, transcriptional regulation, cellular localization, and transport. Herein, for the first time, we report the involvement of the host SUMOylation pathway in the process of infection of *Leishmania donovani*, a causative agent of visceral leishmaniasis. Our data revealed that infection of *L. donovani* to the host macrophages leads to upregulation of SUMOylation pathway genes and downregulation of a deSUMOylating gene, SENP1. Further, to confirm the effect of the host SUMOylation on the growth of *Leishmania*, the genes associated with the SUMOylation pathway were silenced and parasite load was analyzed. The knockdown of the SUMOylation pathway led to a reduction in parasitic load, suggesting the role of the host SUMOylation pathway in the disease progression and parasite survival. Owing to the effect of the SUMOylation pathway in autophagy, we further investigated the status of host autophagy to gain mechanistic insights into how SUMOylation mediates the regulation of growth of *L. donovani*. Knockdown of genes of host SUMOylation pathway led to the reduction of the expression levels of host autophagy markers while promoting autophagosome–lysosome fusion, suggesting SUMOylation-mediated autophagy in terms of autophagy initiation and autophagy maturation during parasite survival. The levels of reactive oxygen species (ROS) generation, nitric oxide (NO) production, and pro-inflammatory cytokines were also elevated upon the knockdown of genes of the host SUMOylation pathway during *L. donovani* infection. This indicates the involvement of the SUMOylation pathway in the modulation of protective immune responses and thus favoring parasite survival. Taken together, the results of this study indicate the hijacking of the host SUMOylation pathway by *L. donovani* toward the suppression of host immune responses and facilitation of host autophagy to potentially facilitate its survival. Targeting of SUMOylation pathway can provide a starting point for the design and development of novel therapeutic interventions to combat leishmaniasis.

## Introduction

Visceral leishmaniasis (VL) also known as kala-azar, is one of the most neglected tropical diseases caused by an intracellular obligate protozoan parasite, *Leishmania donovani* (Badaro et al., 1986). VL is the most severe form of leishmaniasis; manifests symptoms like irregular bouts of fever, lack of appetite, weight loss, enlargement of the spleen and liver, and anemia; and may cause death if left untreated (Khusro and Aarti, 2017; Burza et al., 2018). It remains one of the top parasitic diseases with the potential of an outbreak and maximum mortality with an estimated 700,000 to 1 million new cases annually (WHO, 2022). *Leishmania* parasites complete their life cycle in two different hosts and exist in two forms: the infective stage promastigotes in the phlebotomine sand fly and amastigotes, and the replicative and disease-causing form in the mammalian host (Dostálová and Volf, 2012). Macrophages are considered one of the critical host cells infected by *L. donovani* promastigotes where these promastigotes differentiate into amastigotes. The early responses from the macrophages and other antigen-presenting cells (APCs) to encounter the infection include the production of IL-12, which leads to the induction of IFN-γ from Th1 cells (Kawamoto et al., 2000). This mechanism induces the production of reactive oxygen species (ROS) and nitric oxide (NO), the major microbicidal properties of macrophages that effectively eliminate intracellular parasites (Nandan et al., 1999; Kaye and Aebischer, 2011). However, despite the initiation and induction of innate and adaptive immune responses, the cumulative effect of multiple factors suppresses host protective immune responses that allow the parasite to establish long-lasting survival and the infection (Sharma and Singh, 2009). For this, the major mechanisms that are altered by the *Leishmania* parasite include the downregulation of oxidative stress-mediated phagocytosis process of macrophages (Kima, 2007; Olivier et al., 2012; Carneiro et al., 2018) and activation of IL-10 secretion (Ribeiro-de-Jesus et al., 1998; Bhattacharyya et al., 2001). Activated IL-10 inhibits the production of many pro-inflammatory cytokines including TNF-α (Gautam et al., 2011) and IL-32γ (Galdino et al., 2014) to promote the uncontrolled spread of the parasite. Another important mechanism employed by the *Leishmania* parasites to evade immune response is the induction of host autophagy. The expression of Beclin-1, Atg5, and LC3-II was induced upon *Leishmania* infection, thus suppressing T-cell responses and promoting parasite survival (Mitroulis et al., 2009; Cyrino et al., 2012; Crauwels et al., 2015; Frank et al., 2015; Pitale et al., 2019).

Post-translational modifications play an important role in the regulation of various disease progressions. SUMOylation, one of the post-translational modifications, is essential for many cellular functions by regulating protein–protein interactions, protein activity, and the localization of the protein (Hickey et al., 2012; Ranjha et al., 2019). The process involves the modification of the lysine residue of a substrate protein by the covalent linkage of a polypeptide, SUMO (Small Ubiquitin-like MOdifier). This SUMO moiety is transferred to a lysine residue usually found within a consensus motif ΨKxE/D (Hay, 2005). The machinery is enzymatically similar to ubiquitylation but mechanically different as having its E1 SUMO enzyme (the AOS-1/UBA2 heterodimer), an E2 SUMO enzyme (UBC9), and E3 SUMO enzymes, which enhance SUMO conjugation of specific targets (Geiss-Friedlander and Melchior, 2007). SUMOylation is a continuous and reversible mechanism regulated by SUMO-specific cysteine proteases (SENPs) (Guo and Henley, 2014, Kunz et al., 2018). In mammals, six SENP isoforms are present. Among all 6 SENPs, SENP1 has extensively been reported as the only protease with higher specificity and isopeptidase activity toward SUMO-1 (Kolli et al., 2010), while other SENPs have proteolytic cleavage preference for SUMO-2/3 (Gong and Yeh, 2006; Huang et al., 2009; Dou et al., 2010; Bawa-Khalfe et al., 2012). SENP1 also has a proteolytic activity for SUMO-2/3 substrates. Also, SENP1 regulates various signaling mechanisms in the macrophages (Qiu et al., 2017; Yu et al., 2017). SUMOylation plays an important role in various cellular processes including DNA repair (Sarangi et al., 2014), several types of cell death (Cho et al., 2015; Yang et al., 2019; Gâtel et al., 2020; Li et al., 2020; Liu et al., 2020), oxidative stress (Akar and Feinstein, 2009; Pandey et al., 2011; Stankovic-Valentin and Melchior, 2018), and the regulation of immune responses during many bacterial and viral infections (Ribet et al., 2010; Fritah et al., 2014; Sidik et al., 2015). SUMO pathway obstructs the activation of innate immune responses with its inhibitory effect on various immune regulators that include IRFs (Nakagawa and Yokosawa, 2002; Han et al., 2008; Kubota et al., 2008), STAT proteins (Begitt et al., 2011; Van Nguyen et al., 2012), nuclear factor-κB (NF-κB) (Liu et al., 2012), and NF-κB inhibitor-α (IκBα) (Desterro et al., 1998). SUMOylation of STAT1 by a single Lys703 residue induces IFN-γ signaling (Ungureanu et al., 2003).

SUMOylation has been reported to be exploited by different viral proteins, thus aiding viral activity and replication by interfering with the host cellular environment (Marcos-Villar et al., 2011; Chang et al., 2016). Considering the diverse roles of SUMOylation in modulating immunity and immune responses, it comes as no surprise that bacteria, viruses, and parasites have developed mechanisms and strategies to hijack the SUMOylation pathway that contributes to the suppression of innate immune signaling. As SUMOylation is the common key to regulating many downstream pathways, we propose that this novel pathway may be targeted for therapeutic intervention for leishmaniasis. Here, we are reporting the role of host SUMOylation in regulating various defense and immune responses from macrophages during leishmaniasis. Our results indicate that the host SUMOylation pathway favors the infection and growth of *L. donovani* in the macrophages through the modulation of host survival mechanisms and inflammatory immune responses. This study would aid to identify better strategies for the development of an effective vaccine or drug candidate.

## Material and Methods

### Parasite and Cell culture

*L. donovani* Bob promastigotes were cultured in M199 medium (Gibco, Grand Island, NY, USA) supplemented with 10% heat-inactivated fetal bovine serum (FBS) (Gibco) and 10 µg/ml of gentamicin (Life Technologies, Carlsbad, CA, USA) at 26°C. Metacyclic stage parasites at 20 multiplicity of infection (MOI) were used for infection. THP-1 cells were purchased from National Centre for Cell Science (NCCS, Pune, India) and were maintained in RPMI-1640 (Gibco) media supplemented with 10% FBS (Gibco), 2 mM of l-glutamine, 10 mM of HEPES buffer, 20 mM of sodium bicarbonate, 1 mM of sodium pyruvate, and penicillin/streptomycin (10,000 units/ml) at 37°C in a humidified incubator with 5% CO_2_. Phorbol 12-myristate 13-acetate (PMA) (Sigma, St. Louis, MO, USA) at a concentration of 50 ng/ml for 24 h was used to differentiate the monocytes into macrophages.

### siRNA Transfection

For siRNA transfections, all siRNAs—SUMO-1 (sc-29498), SUMO-2/3 (sc-37167), AOS-1 (sc-60174), UBA2 (sc-61740), UBC9 (sc-36773), SENP1 (sc-44449), and control siRNA (sc-37007)—were procured from Santa Cruz (Dallas, TX, USA). Briefly, THP-1 macrophages were transfected with 60 pmol of siRNA in Opti-MEM medium (Gibco) using the Hiperfect transfection reagent (Qiagen, Valencia, CA, USA) as per the manufacturer’s protocol. After 5 h of transfection, cells were supplemented with the complete media containing 20% FBS and further incubated for 36 h. Knockdown efficiency was measured by qRT-PCR and Western blotting. For infection studies, these transfected THP-1 macrophages were infected with purified metacyclic *L. donovani* promastigotes at 20 MOI.

### qRT-PCR for Gene Expression Analysis

Total RNA from infected or transfected macrophages was isolated at an appropriate time point using the TRIzol reagent (Invitrogen, Grand Island, NY, USA) and quantified using a Nanodrop ND-1000 spectrophotometer (Thermo Fisher Scientific, Waltham, MA, USA). One microgram of total RNA was used for cDNA synthesis, done by First Strand cDNA Synthesis Kit (Thermo Fisher Scientific, USA), as per manufacturer’s instructions, using random hexamer primers. PCRs were carried out in Applied Biosystems, Real-Time PCR System (ABI, Foster City, CA, USA) using PowerUp SYBR Green PCR Master Mix (Thermo Fisher Scientific, USA). Primer sequences for *SUMO-1*-Forward 5′-CATTGGACAGGATAGCAGTGAG-3′, Reverse 5′-CTCTGACCCTCAAAGAGAAACC-3′; *SUMO-2/3*-Forward 5′-AGAGGCATACACCACTTAGTAAAC-3′, Reverse 5′-TCTGCTGTTGGAACACATCAA-3′; *AOS-1*-Forward 5′-ACGACCTCCGACTACTTTCT-3′, Reverse 5′-GCCAAATCTTGAGTTCACTTGG-3′; *UBA2*-Forward 5′-GGCAGCTGATGTTCCTCTTATT-3′, Reverse 5′-CACTCATAACACTCGGTCACAC-3′; *UBC9*-Forward 5′-CAGTGTGCCTGTCCATCTTAG-3′, Reverse 5′-CTTGAGCTGGGTCTTGGATATT-3′; *SENP1*-Forward 5′-TAGTGAACCACAACTC CGTATTC-3′, Reverse 5′-ATGTCCTTGCCTGGAAGATAAA-3′; *IL-10*-Forward 5′ –TTAAGGGTTACCTGGGTTGC-3′, Reverse 5′-TGAGGGTCTTCAGGTTCTCC-3′; *IL-12*-Forward 5′-ATGCCCCTGGAGAAATGGTG-3′, Reverse 5′-GGCCAGCATCTCCAAA CTCT-3′; *IL-32γ*-Forward 5′-AGGCCCGAATGGTAATGCT-3′, Reverse 5′-CCACAGTG TCCTCAGTGTCACA-3; *TNF-α*-Forward 5′-CCTCTCTCTAATCAGCCCTCTG-3′, Reverse 5′-GAGGACCTGGGAGTAGATGAG-3′; *RNU6AP*-Forward 5′-GGCCCAGCA GTACCTGTTTA-3′, Reverse 5′-AGATGGCGGAGGTGCAG-3′. Amplification at 50°C for 2 min followed by 40 cycles at 95°C for 15 s, 60°C for 30 s, and 72°C for 1 min was the thermal profile used for the real-time PCR. Melting curves were generated along with the mean C_T_ values for confirmation of the generation of a specific PCR product. Amplification of RNU6AP (RNA, U6 small nuclear 1; THP-1 cells) was used as the internal control for normalization. The results were expressed as fold change of control [uninfected samples (RNU6AP)] using the 2^−ΔΔCT^ method. Each experiment was done in triplicates and repeated three times.

### Cell Viability Assay

MTT assay was done to assess the effect of siRNAs on THP-1 cell viability. MTT [3-(4,5-dimethyl-2-thiazolyl)-2,5-diphenyltetrazolium bromide] dye solution (Sigma-Aldrich, USA) [5 mg of MTT in 1 ml of phosphate-buffered saline (PBS)] was diluted 1:10 in RPMI medium. For the MTT assay, THP-1 cells (10,000 cells/100 µl) were seeded in each well of 96-well flat-bottom plates. After the differentiation of monocytes into macrophages, THP-1 macrophages were transfected with respective siRNAs as described above. After 36 h, the MTT assay was performed as per the manufacturer’s protocol. Briefly, untransfected and transfected THP-1 macrophages were incubated with MTT dye solution for 2 h at 37°C, and then the stopping buffer (5% formic acid in isopropanol) was added to stop the reaction at 37°C for 20 min. Absorption was then measured at 570 nm, and the percentage of cell viability was calculated. Each experiment was done in triplicates and repeated three times.

### Quantification of *Leishmania donovani* by Confocal Microscopy

THP-1 cells at 0.5 × 10^6^ were seeded on 18-mm-diameter round coverslips coated with poly-l-lysine (Sigma) and were stimulated with 50 ng/ml of PMA to differentiate into macrophages for 24 h. After the incubation, the coverslips were washed with RPMI-1640 medium to remove the non-adherent cells. Then, adherent THP-1 macrophages were transfected with specific siRNAs for 36 h followed by the infection with metacyclic *L. donovani* promastigotes at 20 MOI. After 6 h of infection, cells were washed thrice with RPMI medium to remove the non-phagocytosed promastigotes. Infected macrophages were further incubated for 24 h. After incubation, the coverslips were washed with PBS followed by the fixation with chilled methanol and then stained with propidium iodide (500 nM in 2× SSC buffer) (Ramu et al., 2019). A minimum of 100 macrophages was counted per coverslip under the confocal microscope to determine the number of resident amastigotes. Confocal imaging was performed with Olympus Fluoview FV1000 with 60× objective magnifications, using an excitation/emission wavelength of 535/617 nm.

### Immunoblotting

Primary antibodies used in Western blotting with dilutions were as follows: SUMO-1 (Santa Cruz #sc-5308; 1:1,000), SUMO-2/3 (Santa Cruz #sc-393144; 1:1,000), UBA2 (Santa Cruz #sc-376305; 1:1,000), AOS-1 (Santa Cruz #sc-398080; 1:1,000), UBC9 (Santa Cruz #sc-271057; 1:1,000), SENP1 (Santa Cruz #sc-271360; 1:1,000), β-actin (Santa Cruz #sc-8432; 1:1,000), Beclin-1 (CST, Danvers, MA, USA; #3495T, 1:1,000), Atg5 (CST #12994T, 1:1,000), and LC3A/B (CST #4108S, 1:1,000). Goat anti-mouse horseradish peroxidase (HRP)-conjugated secondary antibody (1:5,000) was purchased from Invitrogen (Carlsbad, CA, USA). Goat anti-rabbit HRP-conjugated secondary antibody (1:2,500) was purchased from ABclonal (Woburn, MA, USA). Briefly, differentiated and transfected THP-1 macrophages were infected with *L. donovani* promastigotes at 20 MOI for 48 h. Cell lysates were prepared by using radioimmunoprecipitation assay (RIPA) lysis buffer (G-Biosciences, St. Louis, MO, USA) with the addition of a protease inhibitor cocktail (Roche, Basel, Switzerland). An equal amount of cell lysates was loaded and separated through sodium dodecyl sulfate–polyacrylamide gel electrophoresis (SDS-PAGE). The proteins were transferred onto the nitrocellulose immunoblot membrane (Bio-Rad, Hercules, CA, USA). The membrane was blocked in 5% bovine serum albumin (BSA) in 1× PBS containing 0.05% Tween 20 (Sigma) (wash buffer) overnight at 4°C, then washed thrice with wash buffer, and further incubated with primary antibodies for 2 h at room temperature following three washes and incubation with HRP-conjugated secondary antibody for another 1 h at room temperature. After three washes, blots were visualized with a densitometric enhanced chemiluminescence (ECL) kit (Bio-Rad) on the ChemiDoc Imaging System (Bio-Rad). Data were analyzed using ImageJ software.

### Confocal Analysis

THP-1 cells measuring 0.5 × 10^6^ were seeded on 18-mm-diameter round coverslips coated with poly-l-lysine (Sigma) and were stimulated with 50 ng/ml of PMA to differentiate into macrophages for 24 h. THP-1 macrophages were transfected with specific siRNAs for 36 h followed by the infection with metacyclic *L. donovani* promastigotes at 20 MOI for 24 h. Cells were washed with RPMI followed by fixation with 2% paraformaldehyde (PFA) for 15 min. Cells were washed with 1× PBS and were permeabilized with a permeabilization buffer (0.1% BSA and 0.2% saponin in 1× PBS). Cells were washed with washing buffer (0.1% BSA and 0.1% saponin in 1× PBS) followed by the incubation of both primary antibodies for LAMP-1 (CST #9091S) and MAP LC3α/β (Santa Cruz #sc-398822) together for 0.5 h at room temperature. Following washes, secondary antibodies (Alexa Fluor 488 goat anti-rabbit IgG (Invitrogen #A11008) for LAMP1 and Alexa Fluor 546 goat anti-mouse IgG (Invitrogen #A11030) for LC3) were introduced to the cells for 20 min at room temperature. Cells were washed, and the coverslips were mounted on the slides with a mounting medium, fluoroshield with DAPI (Sigma). The colocalization of both antibodies was acquired and analyzed under confocal microscopy. Confocal imaging was performed with Olympus Fluoview FV1000 with 60× objective magnifications. Colocalization was measured by Pearson’s correlation coefficient, with values ranging between −1 and +1.

### Measurement of Reactive Oxygen Species

THP-1-differentiated human macrophages were transfected with siRNAs for 36 h in 96-well plates (Corning, New York, NY, USA; #CLS3603). Macrophages were infected with *L. donovani* promastigotes at 20 MOI for 30- and 60-min time points. Thirty minutes before completing the incubation period, cells were loaded with 10 µM of DCFH-DA according to the manufacturer’s instructions (Abcam, Cambridge, UK; #ab113851). At the end of the incubation period, cells were immediately analyzed for ROS generation levels by fluorometry with excitation/emission at 485/535 nm.

### Measurement of Nitric Oxide

PMA-differentiated THP-1 macrophages were transfected with siRNAs for 36 h followed by the infection with 20 MOI of *L. donovani* promastigotes along with the co-stimulation of lipopolysaccharide (LPS) (100 ng/ml) and hIFNγ (20 ng/ml). At 24 h post-infection, the supernatant was collected to estimate the NO level (in the form of nitrite) using the Griess reagent kit (Invitrogen #G-7921) as per the manufacturer’s instructions.

### Immunoassay for Cytokines

siRNA-transfected THP-1 macrophages were infected with 20 MOI of *L. donovani* and stimulated with LPS (100 ng/ml). After 24 h of infection, the supernatant was collected and centrifuged for further processing. The cytokine level for human IL-12p40 (430704), human TNF-α (430204), human IFN-γ (430104), and human IL-10 (430601) was measured using commercial ELISA kits from BioLegend (San Diego, CA, USA), according to the manufacturer’s instructions.

### Statistical Analysis

Data were presented as the mean ± SD. Each experiment was repeated three times in separate sets. All graphs generated and the related statistical analyses were performed using GraphPad Prism (GraphPad Software, La Jolla, CA, USA). Statistical significance was quantified using the unpaired t-test with Welch’s correction. Significance was reached with *p*-values <0.05. *p*-Values were shown as *p < 0.05, **p < 0.01, ***p < 0.001, and ****p < 0.0001.

## Results

### *Leishmania donovani* Infection Induces the Expression Level of Genes Involved in the Host SUMOylation Process

**Figure 1:**
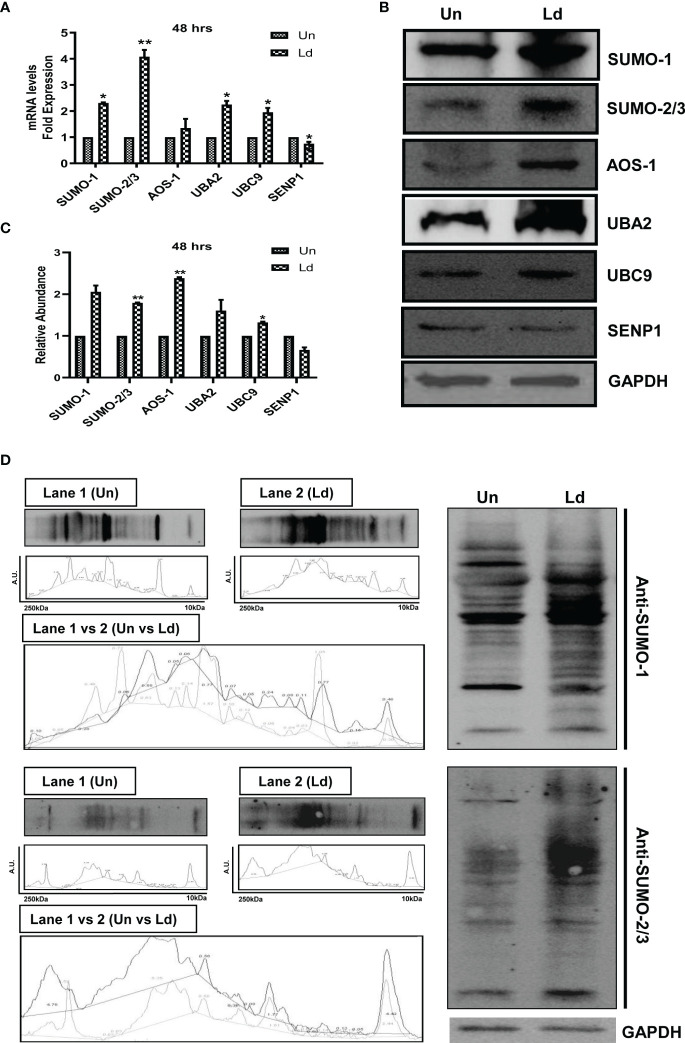
Induced expression of genes involved in host SUMOylation pathway upon *Leishmania donovani* infection. PMA-differentiated THP-1 macrophages were infected with metacyclic stage *L. donovani* promastigotes at 20 MOI for 48 (h) Uninfected (Un) and *L. donovani*-infected (Ld) cells were processed for different evaluations. **(A)** RNA was isolated from uninfected and infected macrophages at 48 h post-infection, and expression analysis of genes of the host SUMOylation pathway was performed by quantitative real-time PCR (qRT-PCR). *p*-Value was calculated based on Student’s unpaired 2-tailed t-test comparing uninfected macrophages to infected macrophages (*p < 0.05 and **p < 0.01). **(B)** Cytoplasmic extracts of uninfected and infected macrophages were analyzed by Western blotting to check the expression level of SUMO-1, SUMO-2/3, AOS-1, UBA2, UBC9, and SENP1 at 48 h post-infection. GAPDH was used as the loading control. Band intensities were calculated using ImageJ software. Data from one of three experiments are shown. **(C)** The graph represents the relative intensity of the bands calculated by ImageJ software at 48 h post-infection; *p = 0.028 for UBC9; **p = 0.0043 and 0.0073 for SUMO-2/3 and AOS-1, respectively. *p*-Value was calculated based on an unpaired t-test with Welch’s correction comparing uninfected macrophages to *L. donovani*-infected macrophages (Un vs. LD). **(D)** Cytoplasmic extracts of uninfected and infected macrophages were analyzed by Western blotting to check the expression of overall SUMOylated proteins at 48 h post-infection. GAPDH was used as the loading control. The band intensities of each lane were analyzed by ImageJ software, and the values concerning band intensity are labeled on the peaks. PMA, phorbol 12-myristate 13-acetate; MOI, multiplicity of infection.

First, we investigated the effect of *L. donovani* infection on the expression of different genes of the host SUMOylation pathway. As shown in **Figure 1A**, infection of PMA-differentiated THP-1 macrophages with *L. donovani* promastigotes significantly upregulated the expression levels of *SUMO-1*, *SUMO-2/3*, *UBA2*, and *UBC9* by ≥2-fold, while a significant downregulation of *SENP1* was observed at 48 h post-infection at the transcript level. We also analyzed the expression of these genes in *L. donovani*-infected macrophages at the protein level (**Figure 1B**). The results confirmed the significant upregulation of a minimum of 1.5-fold in the expression levels of SUMO-2/3, SUMO-activating E1 enzymes (AOS-1), and SUMO conjugating E2 enzyme (UBC9) at 48 h post-infection compared to uninfected macrophages (**Figure 1C**). These results indicate the upregulation of the SUMOylation and the downregulation of the deSUMOylation process in the human macrophages following infection by *L. donovani*. We also investigated the effect of *L. donovani* infection on the overall SUMOylation in the macrophages. As shown in **Figure 1D**, a significant upregulation of the expression profile of SUMOylated proteins by both SUMO-1 and SUMO-2/3 was observed at 48 h post-infection. This result indicates an upregulation of SUMOylation of the proteins in *Leishmania*-infected macrophages that might determine the fate of parasites in the macrophages.

### Knockdown of the Genes Involved in the SUMOylation Pathway Results in the Reduced Parasitic Load in Macrophages

**Figure 2:**
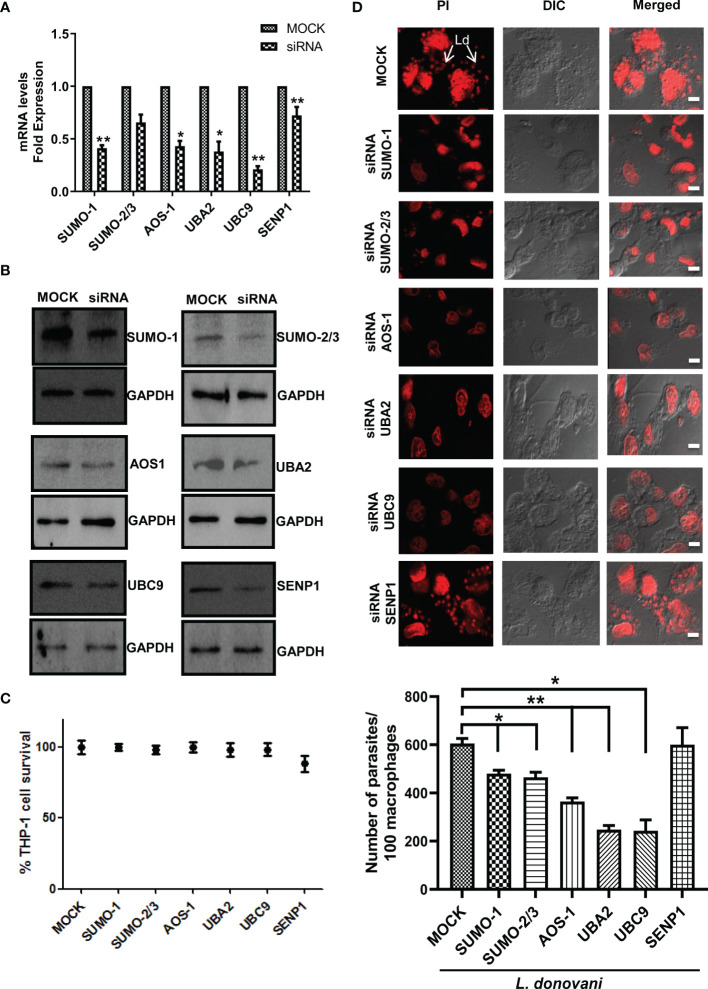
Host SUMOylation pathway favors the growth of *Leishmania donovani* in macrophages. PMA-differentiated THP-1 macrophages were transiently transfected with siRNA against specific genes for 36 h MOCK here represents the transfection with a control siRNA. **(A)** Total RNA was enriched using TRIzol (Qiagen). mRNA levels of transfected macrophages with target genes compared to MOCK were quantified by qRT-PCR. *p = 0.02, 0.03, and 0.04 for MOCK vs. *SUMO-1*, *AOS-1*, and *UBA2*, respectively; **p = 0.0012 and 0.01 for MOCK vs. *UBC9* and *SENP1*, respectively. **(B)** Cell lysates of transfected macrophages were used to check the transfection efficiency by Western blotting. GAPDH was used as the loading control for each set separately. **(C)** Effect of transfection on cell viability measured by MTT assay. THP-1 macrophages were transfected with specific siRNAs and control siRNA (MOCK) for 36 (h) No significant changes in cell viability were observed upon the transfection of siRNAs against target genes compared to MOCK. **(D)** PMA-differentiated macrophages were transfected with specific siRNAs followed by *L. donovani* infection for 6 (h) Macrophages were stained with propidium iodide after 24 h post-infection, and parasitic load in the infected macrophages was calculated under confocal microscopy. Scale bar, 5 µm. Data from one of three experiments are shown. Parasite load was counted in 100 macrophages. Statistical significance was quantified using the unpaired t-test with Welch’s correction, *p = 0.0286, 0.0222, and 0.0266 for MOCK vs. *SUMO-1*, *SUMO-2/3*, and *UBC9*, respectively; **p = 0.0087 and 0.0034 for MOCK vs. *AOS-1* and *UBA2*, respectively. PMA, phorbol 12-myristate 13-acetate.

To further investigate the involvement of SUMOylation in leishmaniasis progression, we inhibited the expression level of these genes by using siRNA. The transfection efficiency was measured at both transcript and protein levels. The results revealed a significant transfection efficiency with ~50% reduction in the expression level of *SUMO-1* and SUMO-activating E1 enzymes (*AOS-1* and *UBA2*) and ~75% reduction in the expression level of *UBC9* at the transcript level (**Figure 2A**); on the other hand, at the protein level, a significant reduction of ~50% was observed for the expression of SUMO-1, SUMO-2/3, SUMO-activating E1 enzymes (AOS-1 and UBA2), SUMO-conjugating E2 enzyme (UBC9), and SENP1 compared to MOCK (**Figure 2B**). We also evaluated whether the process of a knockdown of the genes in macrophages affects the cell viability by performing an MTT assay and found no significant changes in the cell viability (**Figure 2C**). To investigate the role of genes involved in host SUMOylation and deSUMOylation process in the disease progression of *Leishmania*, we silenced the genes followed by *L. donovani* infection and monitored the survival of *L. donovani* amastigotes inside the macrophages. As shown in **Figure 2D**, the knockdown of *SUMO-1*, *SUMO-2/3*, SUMO-activating E1 enzymes (*AOS-1* and *UBA2*), and SUMO-conjugating E2 enzyme (*UBC9*) significantly reduced the number of amastigotes in the human macrophages as compared to control infected macrophages. Interestingly, the reduction was more than 50% upon the knockdown of *UBA2* and *UBC9*. Inhibition of SUMO-activating and SUMO-conjugating enzymes could lead to the complete inhibition of the SUMOylation process. That might lead to the reduction of protein SUMOylation essentially required for *L. donovani* survival. However, no change in intracellular parasite load was observed upon the knockdown of the deSUMOylating gene *SENP1* followed by *L. donovani* infection. Since there are six different SENPs in mammals, this could be due to redundancy among them or that downregulation of SENP1 may not alter the SUMOylation status of proteins that directly or indirectly determine parasite load. These results point toward the essentiality of the host SUMOylation process necessitated by *L. donovani* for its survival.

### Host SUMOylation Process Regulates the Initiation of Host Autophagy During *Leishmania donovani* Infection

**Figure 3:**
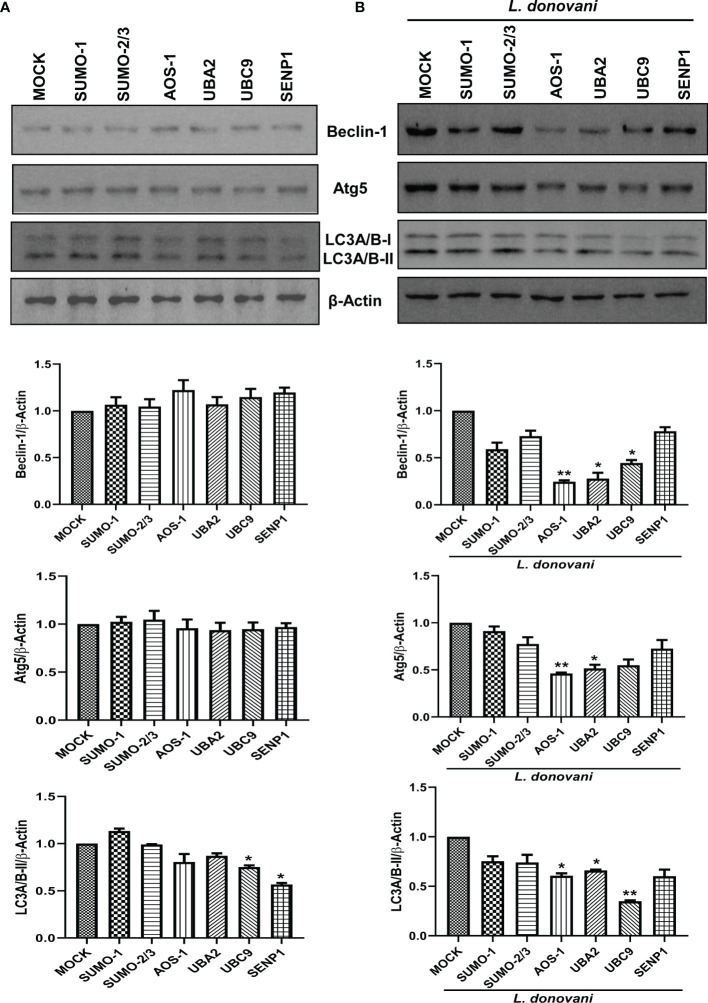
Host SUMOylation regulates the growth of *Leishmania donovani* by modulating autophagy initiation. **(A)** 1 × 10^6^ THP-1 cells were differentiated into macrophages and were transfected with the specific siRNAs **(B)** followed by *L. donovani* infection. MOCK here represents the transfection with a control siRNA. Cell lysates were prepared at 48 h post-infection and were processed to check the expression level of different autophagy markers Beclin-1, Atg5, and LC3A/B. Data from one of three experiments are shown. Band intensities were quantified by ImageJ software and were plotted in GraphPad Prism 8. Statistical significance was quantified using the unpaired t-test with Welch’s correction, **(A)** *p = 0.03 and 0.016 for MOCK vs. *UBC9* and *SENP1*, respectively, for the expression of LC3A/B-II. **(B)** *p = 0.038 and 0.018 for MOCK vs. *UBA2* and *UBC9*, respectively, and **p = 0.01 for MOCK vs. *AOS-1* for the expression of Beclin-1; *p = 0.035 and 0.043 for MOCK vs. *UBA2* and *UBC9*, respectively, and **p = 0.01 for MOCK vs. *AOS-1* for the expression of Atg5; *p = 0.027 and 0.011 for MOCK vs. *AOS-1* and *UBA2*, respectively, and **p = 0.006 for MOCK vs. *UBC9* for the expression of LC3A/B-II.

To investigate the mechanisms employed by these genes to favor the parasite growth, we further characterized their function in host-mediated survival strategy. In recent decades, various studies have consistently reported the induction of host autophagy by all species of *Leishmania*, *in vitro* and *in vivo* (Mitroulis et al., 2009; Cyrino et al., 2012; Frank et al., 2015; Matte et al., 2016; Franco et al., 2017; Dias et al., 2018; Pitale et al., 2019). Inhibition of autophagy also leads to reduced intracellular *L. donovani* survival (Thomas et al., 2018). In this connection, we next studied the role of host SUMOylation and deSUMOylation processes in the regulation of host autophagy. We monitored the expression of three autophagy markers: i) Beclin-1, which binds to Atgs essential to form a phagophore; ii) Atg5, which conjugates to Atg12 crucial for the conjugation of LC3 to phagophore; and iii) LC3, which yields LC3-II upon proteolytic cleavage and lipidation required to form the autophagosome. It is well-reported that the expression of these genes was induced during *Leishmania* infection (Cyrino et al., 2012; Frank et al., 2015; Thomas et al., 2018; Pitale et al., 2019). As shown in **Figure 3A**, downregulation of genes from the host SUMOylation pathway leads to no significant change in the expression levels of Beclin-1 and Atg5. However, the knockdown of *UBC9* and *SENP1* significantly reduced the expression of LC3A/B-II. To study the role of host SUMOylation in the regulation of autophagy upon *L. donovani* infection, we monitored the expression levels of autophagy markers in *L. donovani*-infected macrophages. As shown in **Figure 3B**, upon *L. donovani* infection, knockdown of *SUMO-1*, SUMO-activating E1 enzymes (*AOS-1* and *UBA2*), and SUMO-conjugating E2 enzyme (*UBC9*) significantly reduced the expression level of Beclin-1 with ~3-fold decrease; similarly, a significant reduction in the expression level of Atg5 and LC3A/B-II was observed upon the knockdown of SUMO-activating E1 enzymes (*AOS-1* and *UBA2*) and SUMO-conjugating E2 enzyme (*UBC9*) with ~2-fold decrease. However, no significant changes were observed upon the knockdown of *SUMO-1*, *SUMO-2/3*, and *SENP1* compared to MOCK-infected cells. Interestingly, upon comparing the expression levels of autophagy markers from uninfected to infected macrophages, we observed that knockdown of SUMOylation pathway genes markedly reduced the levels of autophagy markers in infected macrophages compared to uninfected macrophages. These results collectively indicate that *L. donovani* infection leads to the modulation of the host SUMOylation process and thereby regulates autophagy.

### Host SUMOylation Modulates the Phagolysosome Fusion During the Infection of *Leishmania donovani*

**Figure 4:**
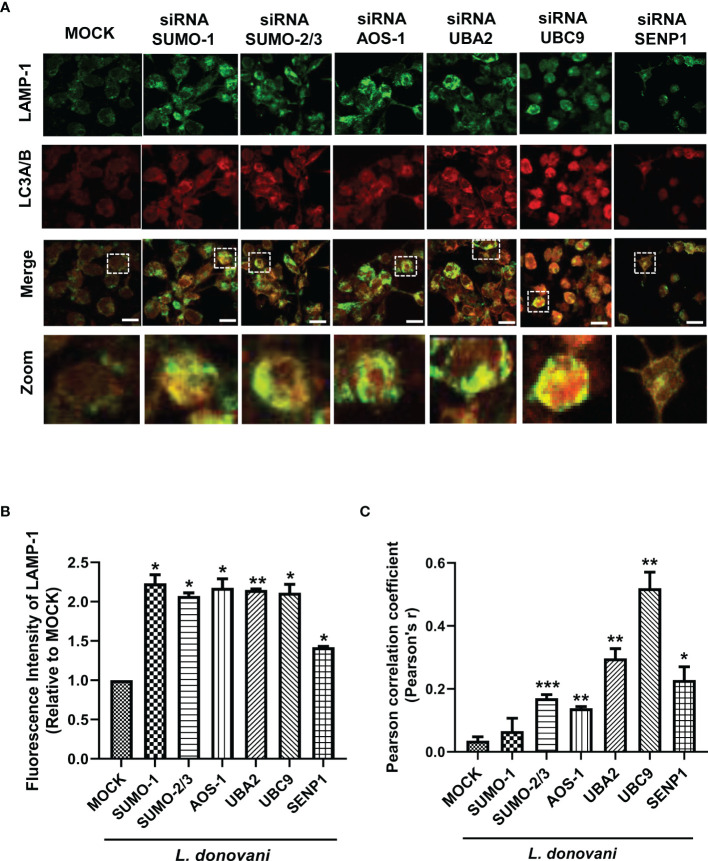
Host SUMOylation favors the growth of *Leishmania donovani* by modulating autophagy maturation. Colocalization studies of autophagosome marker, LC3A/B, and lysosome marker, LAMP-1, in *L. donovani*-infected macrophages were performed. **(A)** 0.5 × 10^6^ THP-1 macrophages were transfected with specific siRNAs followed by the infection of 20 MOI of *L. donovani* for 24 h MOCK here represents the transfection with a control siRNA. Macrophages were incubated with the antibodies to LAMP-1 and LC3A/B with their respective secondary antibodies (see the *Materials and Methods* section). Images were acquired under confocal microscopy. Here, the green color represents the expression level of LAMP-1, the red color represents the expression level of LC3A/B, and the yellow color represents the colocalization of LAMP-1 and LC3A/B. Scale bar, 10 µm. A ×5 zoom image represents colocalization in a single cell. **(B)** The graph represents the fluorescence intensity measurement of LAMP-1. Statistical significance was quantified using the unpaired t-test with Welch’s correction, *p = 0.04, 0.017, 0.044, 0.043, and 0.011 for MOCK vs. *SUMO-1*, *SUMO-2/3*, *AOS-1*, *UBC9*, and *SENP1*, respectively; **p = 0.004 for MOCK vs. *UBA2*. **(C)** Graphical representation of colocalization measured by Pearson’s correlation coefficient; values range between −1 and +1. A value of +1 indicates a positive and strong correlation, while a value of −1 indicates a negative and weak correlation. Statistical significance was quantified using the unpaired t-test with Welch’s correction, *p = 0.011 for MOCK vs. *SENP1*; **p = 0.0014, 0.0017, and 0.0024 for MOCK vs. *AOS-1*, *UBA2*, and *UBC9*, respectively; ***p = 0.0002 for MOCK vs. *SUMO-2/3*. MOI, multiplicity of infection.

Our data showed that inhibition of host SUMOylation genes reduced the expression level of LC3A/B-II, a marker of autophagosomes upon *L. donovani* infection. For the intracellular killing of a pathogen, the fusion of this autophagosome with lysosome is essential to form a phagolysosome having microbicidal capacity. Therefore, to understand the mechanism of how SUMOylation-mediated autophagy is being regulated during *L. donovani* infection, we further studied its role in phagolysosome fusion, the final and utmost important step of autophagy. PMA-differentiated, PMA-transfected, and PMA-infected THP-1 macrophages were analyzed for the expression of LAMP-1, a lysosome marker, and its colocalization with LC3A/B, a marker of the autophagosome, as observed under a confocal microscope (**Figure 4A**). As shown in **Figure 4B**, a significant upregulation of the LAMP-1 fluorescence intensity was observed upon the downregulation of *SUMO-1*, *SUMO-2/3*, SUMO-activating E1 enzymes (*AOS-1* and *UBA2*), and SUMO-conjugating E2 enzyme (*UBC9*) by ~2-fold and *SENP1* by ~1.5-fold compared to MOCK in *L. donovani*-infected macrophages. Colocalization of LAMP-1 and LC3A/B was measured by Pearson’s correlation coefficient, with values ranging between −1 and +1, and plotted in a graph as shown in **Figure 4C**. Upon infection, knockdown of *SUMO-2/3*, SUMO-activating E1 enzymes (*AOS-1* and *UBA2*), SUMO-conjugating E2 enzyme (*UBC9*), and *SENP1* significantly upregulated the colocalization with 0.17, 0.14, 0.29, 0.52, and 0.23 values of Pearson’s correlation coefficient as compared to MOCK, thus inducing phagolysosome fusion. However, no significant colocalization was observed upon the knockdown of *SUMO-1* (**Figure 4C**). This could be because the target proteins of SUMO-1 might not have any involvement in the regulation of autophagy, as knockdown of *SUMO-1* changes neither the expression of autophagy markers nor the phagolysosome fusion in the infected macrophages. Though upon infection, knockdown of *SUMO-2/3* also does not significantly modulate the expression of autophagy markers but regulates autophagy maturation, suggesting the direct or indirect role of target proteins SUMOylated by SUMO-2/3. Downregulation of SUMO-activating E1 enzymes (*AOS-1* and *UBA2*) or SUMO-conjugating E2 enzyme (*UBC9*) might inhibit the entire pathway, therefore significantly modulating both the autophagy initiation by regulating the expression of autophagy markers and the autophagy maturation by phagolysosome fusion in *L. donovani*-infected macrophages. Surprisingly, the knockdown of *SENP1* also induced phagolysosome fusion. Collectively, the result indicates that the host SUMOylation process facilitates the survival and growth of *L. donovani* by suppressing the phagolysosome fusion, thus providing a niche to *L. donovani* for survival and growth.

### Host SUMOylation Regulates the Reactive Oxygen Species Generation and Levels of Nitric Oxide Production During *Leishmania donovani* Infection

**Figure 5:**
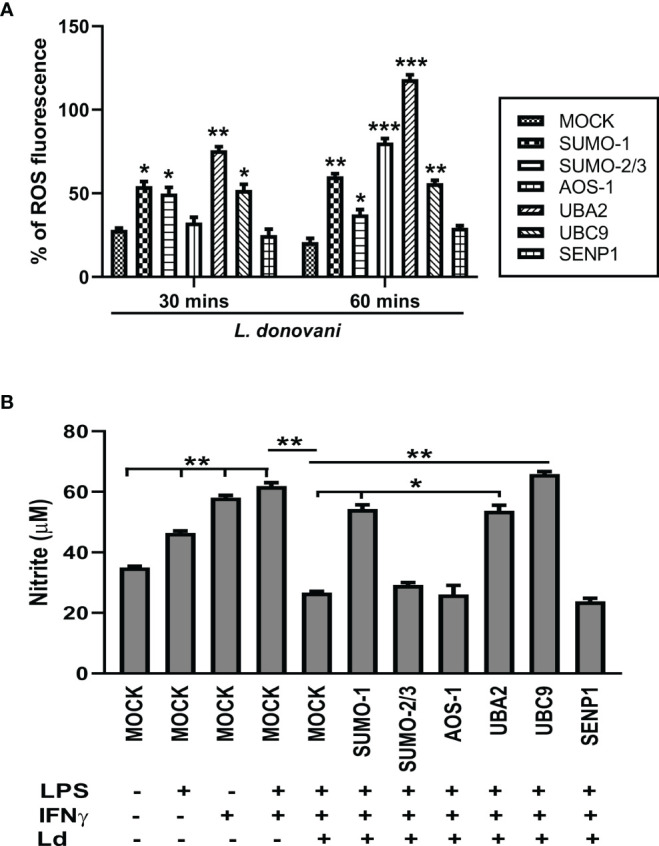
Host SUMOylation favors *Leishmania donovani* survival by modulating the generation levels of ROS and nitric oxide. THP-1-differentiated human macrophages were transfected with siRNAs for 36 h **(A)** in a 96-well plate followed by the infection of *L. donovani* promastigotes at 20 MOI for 30- and 60-min time points. Thirty minutes before completing the incubation period, cells were loaded with 10 µM of DCFH-DA. Samples were immediately analyzed for ROS levels by fluorometry with excitation/emission at 485/535 nm. The graph was plotted in GraphPad Prism 8. Statistical significance was quantified using the unpaired t-test with Welch’s correction, *p = 0.02, 0.05, and 0.04 for MOCK vs. *SUMO-1*, *SUMO-2/3*, and *UBC9*, respectively; **p = 0.005 for MOCK vs. *UBA2* at 30 min post-infection and *p = 0.02 for MOCK vs. *SUMO-2/3*; **p = 0.003 and 0.004 for MOCK vs. *SUMO-1* and *UBC9*, respectively; ***p = 0.001 and 0.0007 for MOCK vs. *AOS-1* and *UBA2*, respectively, at 60 min post-infection. **(B)** Transfected macrophages were infected with *L. donovani* promastigotes at 20 MOI along with the stimulation of LPS (100 ng/ml) and hIFN-γ (20 ng/ml) for 24 h Griess reagent was used to estimate the NO level in the supernatant. The graph was plotted in GraphPad Prism 8. Statistical significance was quantified using the unpaired t-test with Welch’s correction (*p < 0.05 and **p < 0.01). ROS, reactive oxygen species; MOI, multiplicity of infection; LPS, lipopolysaccharide.

ROS and NO are the major strategies of macrophages for regulating cell death and inflammatory responses in encountering the pathogen. In this connection, we further investigated the role of the host SUMOylation process in the regulation of ROS generation and NO production during *L. donovani* infection. Since ROS is an early defense phenomenon, we observed ROS levels in transfected macrophages followed by *L. donovani* infection at 30 and 60 min post-infection. Knockdown of *SUMO-1*, *SUMO-2/3*, and *UBC9* significantly elevated the ROS generation by ~2-fold, while *UBA2* downregulation elevated ROS levels by ~3-fold compared to MOCK at 30 min post-infection. However, knockdown of *AOS1* does not alter the ROS generation in the macrophages at 30 min post-infection. Interestingly, at 60 min post-infection, a significant elevation of ROS generation was observed upon knockdown of *SUMO-2/3* and *UBC9* by ~2-fold, *SUMO-1* by ~3-fold, *AOS-1* by ~4-fold, and *UBA2* by ~6-fold compared to MOCK. No significant change in ROS generation was observed upon knockdown of *SENP1* at 30 and 60 min post-infection (**Figure 5A**). This suggests that during *Leishmania* infection, the host SUMOylation process modulates ROS generation and thus regulates the parasite load. NO is another important component of the macrophage defense mechanism to control *Leishmania* infection. Activation of macrophages by co-stimulation of LPS and IFNγ produces robust NO levels by inducing gene expression of inducible NO synthase (iNOS). Knockdown of *SUMO-1*, *UBA2*, and *UBC9* significantly upregulated the production of NO in infected and LPS- and IFNγ-stimulated macrophages by >2-fold at 24 h post-infection. However, no alteration in NO production was observed upon knockdown of *SUMO-2/3*, *AOS-1*, and *SENP1* in infected macrophages (**Figure 5B**). One possibility is that proteins SUMOylated only by SUMO-2/3 might not regulate NO production in *L. donovani*-infected macrophages. This result also indicates the involvement of the SUMO-1-mediated SUMOylation process to regulate NO production during *L. donovani* infection. Taken together, the results indicate that host SUMOylation favors parasite growth and survival by modulating the levels of ROS generation and NO production of macrophages.

### Host SUMOylation Regulates Pro-Inflammatory Cytokine Production During *Leishmania donovani* Infection

**Figure 6:**
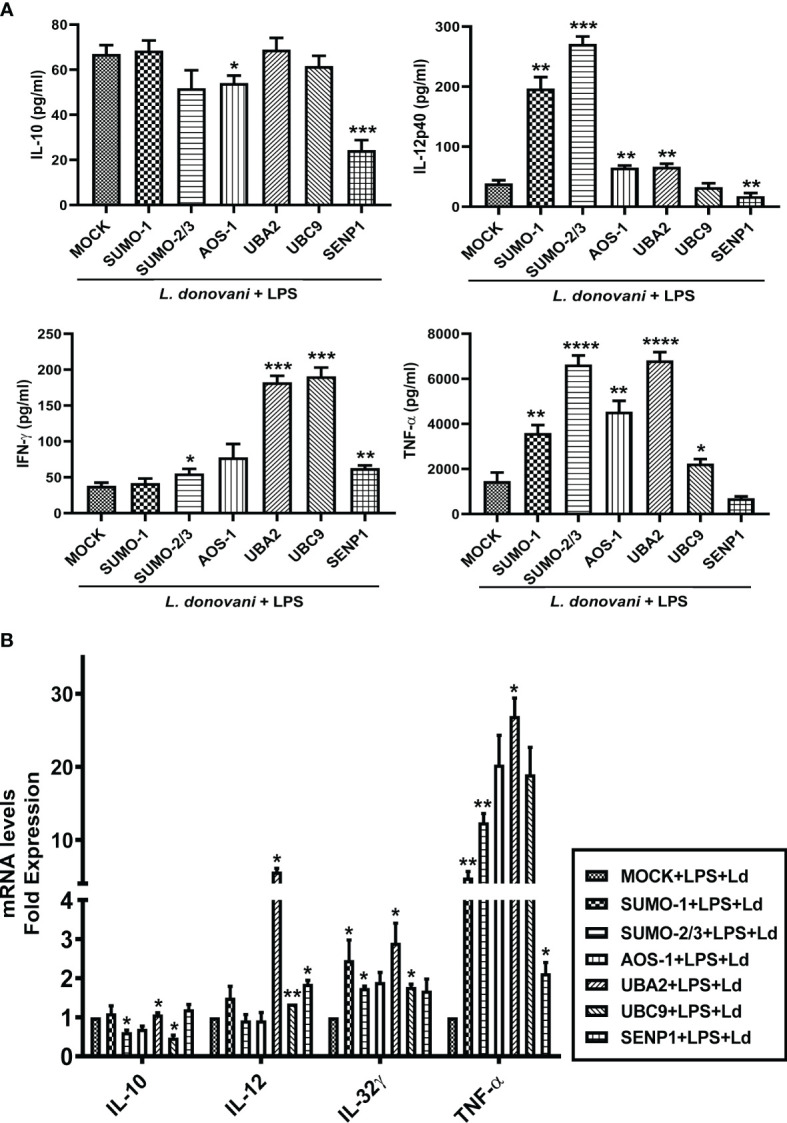
Host SUMOylation promotes the growth of *Leishmania donovani* by suppressing pro-inflammatory cytokines. THP-1 macrophages were transfected with specific siRNAs followed by the infection of *L. donovani* along with or without the stimulation of LPS (100 ng/ml) for 24 h MOCK here represents the transfection with a control siRNA. Culture supernatants were collected to measure different cytokines, while cells were used to check the expression of inflammatory markers at the transcript level. **(A)** Graph represents cytokine levels measured by sandwich ELISA. Statistical significance was quantified using the unpaired t-test with Welch’s correction, *p = 0.012 for MOCK vs. *AOS-1* and ***p = 0.0003 for MOCK vs. *SENP1* for IL-10; **p = 0.0029, 0.0032, 0.0028, and 0.0074 for MOCK vs. *SUMO-1*, *AOS-1*, *UBA2*, and *SENP1*, respectively, and ***p = 0.0002 for MOCK vs. *SUMO-2/3* for IL-12p40; *p = 0.025 for MOCK vs. *SUMO-2/3*, **p = 0.002 for MOCK vs. *SENP1*, and ***p = 0.0002 and 0.0008 for MOCK vs. *UBA2* and *UBC9*, respectively, for IFN-γ; *p = 0.049 for MOCK vs. *UBC9*, **p = 0.0012 for MOCK vs. *AOS-1*, ****p < 0.0001 for *SUMO-2/3* and *UBA2* for TNF-α in LPS-stimulated and Ld-infected macrophages. **(B)** RNA was isolated for gene expression analysis of inflammatory cytokines by quantitative real-time PCR (qRT-PCR). *p = 0.04 for MOCK vs. *SUMO-2/3* or *UBC9* for *IL-10*; *p = 0.04 and 0.05 for MOCK vs. *UBA2* and *SENP1*, respectively; **p = 0.008 for MOCK vs. *UBC9* for *IL-12*; *p = 0.02 for MOCK vs. *SUMO-2/3* and 0.04 for MOCK vs. *SUMO-1* or *UBA2* or *UBC9* for *IL-32γ*; *p = 0.05 and 0.02 for MOCK vs. *UBA2* and *SENP1*, respectively; **p = 0.01 and 0.008 for MOCK *vs*. *SUMO-1* and *SUMO-2/3* for *TNF-α* in LPS-stimulated and Ld-infected macrophages. *p*-Value was calculated based on Student’s unpaired 2-tailed t-test. LPS, lipopolysaccharide.

Cytokines play a crucial role in mediating T-cell responses and host defense mechanisms in macrophages. LPS potently activates macrophages and cytokine signaling by TLR4 stimulation, while *Leishmania* parasites suppress LPS-induced host inflammatory cytokine responses (Lapara and Kelly, 2010). We investigated the effect of perturbation of the host SUMOylation pathway on the pro-inflammatory and anti-inflammatory cytokines from LPS-stimulated macrophages. We found that the knockdown of genes of the host SUMOylation pathway elevated the levels of pro-inflammatory cytokines *IL-12*, *IL-32γ*, and *TNF-α* at the transcript level, while the level of anti-inflammatory cytokine *IL-10* was reduced upon the downregulation of SUMOylation pathway (**Supplementary Figure 1**). We further investigated the role of host SUMOylation and deSUMOylation processes in modulating the cytokines profile using supernatants derived from *L. donovani*-infected and LPS-stimulated macrophages. We first monitored the expression level of an anti-inflammatory cytokine, IL-10. Knockdown of *AOS-1* significantly downregulates the expression level of IL-10 by ~2-fold, while no significant changes were observed upon the knockdown of *SUMO-1*, *SUMO-2/3*, *UBA2*, and *UBC9* in infected macrophages. Surprisingly, a significant reduction of ~2.5-fold was also observed in the expression level of IL-10 upon the knockdown of *SENP1* compared to MOCK. A significant upregulation of IL-12p40, a pro-inflammatory cytokine, was observed upon the knockdown of *SUMO-1* (~6-fold), *SUMO-2/3* (~9-fold), *AOS-1*, and *UBA2* (~2-fold), while knockdown of *SENP1* significantly downregulates the expression level of IL-12p40 by ~2-fold. No significant change in the expression level of IL-12p40 was observed upon the knockdown of *UBC9.* Knockdown of *SUMO-2/3*, *AOS-1*, *UBA2*, and *UBC9* significantly upregulates the expression level of IFN-γ, a pro-inflammatory cytokine; the maximum induction was observed upon the knockdown of *UBA2* and *UBC9* by ~4-fold as compared to MOCK, while knockdown of *SENP1* also upregulates the expression level of IFN-γ to some extent in infected macrophages. Knockdown of *SUMO-1*, *AOS-1*, and *UBC9* significantly upregulates the expression level of TNFα and pro-inflammatory cytokine by ~2-fold; knockdown of *SUMO-2/3* and *UBA2* upregulates the expression level of TNF-α by ~4-fold in infected macrophages. Interestingly, the knockdown of *SENP1* significantly downregulates the expression level of TNF-α (**Figure 6A**). We also monitored the expression level of inflammatory markers upon *L. donovani* infection at the transcript level. A significant reduction was observed in the expression level of *IL-10* at the transcript level upon knockdown of *SUMO-2/3* and *UBC9* by ~2-fold, while no significant changes were observed upon knockdown of other genes compared to MOCK. Knockdown of *UBA2* (~7-fold) and *UBC9* (~2-fold) significantly upregulates the expression level of *IL-12* at the transcript level. The expression level of *IL-32γ*, a pro-inflammatory cytokine, was observed to be significantly upregulated upon knockdown of *SUMO-1*, *SUMO-2/3*, *UBA2*, and *UBC9* by ≥2-fold. Knockdown of *SUMO-1* (~6-fold), *SUMO-2/3* (~12-fold), and *UBA2* (~25-fold) significantly upregulated the expression level of *TNF-α* (**Figure 6B**). Interestingly, the downregulation of the host SUMOylation pathway upregulates the levels of pro-inflammatory cytokines by manifold in the infected macrophages compared to uninfected macrophages. Taken together, these results suggest that the host SUMOylation process favors the infection and survival of *L. donovani* in macrophages by modulating the levels of pro-inflammatory cytokines such as TNF-α, IFN-γ, IL-12p40, and IL-32γ and anti-inflammatory cytokine, IL-10.

## Discussion

SUMOylation has emerged as a host key signaling pathway that has a diverse role in host-mediated immune responses for many viral diseases and cancer biology (Wang et al., 2013; Saul et al., 2015; Xia et al., 2015; Xu et al., 2015; Liu et al., 2016; Wang et al., 2021). To establish the infection and sustain its survival, *Leishmania* parasites successfully manage to evade the host immune mechanism, but information on the host factors or signaling pathways that are modulated by the parasite for the stable infection has not been analyzed in detail. To elucidate the role of host SUMOylation in the pathogenesis of *L. donovani*, we chose to dissect this pathway to observe the roles of different SUMO isoforms, SUMO-E1, and SUMO-E2 enzyme as well one deSUMOylating gene, SENP1. We observed significant changes in these genes’ expression upon *L. donovani* infection. Overall host SUMOylation of many proteins *via* SUMO-1 or SUMO-2/3 was significantly induced upon *L. donovani* infection. This indicates that host SUMOylation might be involved in the regulation of host key downstream signals and subsequent responses to favor *L. donovani* infection. To confirm it, the expression of these genes was inhibited by siRNA-mediated knockdown following *L. donovani* infection. Inhibition of the host SUMOylation pathway at various steps reduced the number of amastigotes inside the macrophages, while no change in the parasite load was observed upon *SENP1* knockdown. The result was correlated to our first data, which validates our hypothesis that host SUMOylation favors the infection and survival of *L. donovani* in the macrophages. Further, we investigated host survival strategies and immune mechanisms modulated by host SUMOylation to regulate *L. donovani* survival.

It is well reported that different species of *Leishmania* induce the expression level of autophagy genes *in vivo* or *in vitro*, thus modulating host autophagy for its advantage (Cyrino et al., 2012; Frank et al., 2015; Thomas et al., 2018; Pitale et al., 2019). Multiple studies have shown that SUMOylation also participates in various cell death mechanisms including autophagy (Kim et al., 2015; Prudent et al., 2015; Mattoscio et al., 2017; Scurr et al., 2017; Lorente et al., 2019). Autophagy is induced upon the depletion of SUMO-1 and thus induced autophagy-mediated cancer cell death (Lorente et al., 2019). Interestingly, there is one report stating that autophagy regulates the SUMO pathway (Mattoscio et al., 2017). Hence, the diverse role of SUMOylation whether negative or positive in the regulation of autophagy solely depends on the substrate protein and their interacting partners. Therefore, we investigated the ability of host SUMOylation in modulating host autophagy for favoring the growth of *L. donovani*. We did not find significant changes in the expression levels of Beclin-1 and Atg5 upon the perturbation of host SUMOylation only. The expression level of LC3A/B-II was reduced upon the knockdown of *UBC9* and *SENP1*. Interestingly, upon *L. donovani* infection, the knockdown of most genes of the host SUMOylation pathway reduced the expression level of Beclin-1, Atg5, and LC3A/B-II. This finding suggests that host SUMOylation favors the growth of *L. donovani* by modulating the initiation of autophagy. The results conforme with the reports (Mitroulis et al., 2009; Thomas et al., 2018; Pitale et al., 2019) that *Leishmania* parasites modulate host autophagy for their welfare. Since the ultimate step of autophagy is the fusion of autophagosomes to lysosomes for the degradation of the internalized cellular components in autolysosomes (Lőrincz and Juhász, 2020), we next investigated the role of host SUMOylation in the autophagy maturation during the infection of *L. donovani*. Our result indicated that blocking genes of host SUMOylation induced the expression level of LAMP-1 and promoted the autophagosome–lysosome fusion. However, knockdown of SENP1 also reduced the expression levels of autophagy markers and induced the level of lysosome marker and autophagosome–lysosome fusion. Collectively, these findings suggest that during *L. donovani* infection, host SUMOylation promotes the initiation of autophagy but suppresses the maturation of autophagy, thus providing a niche for the survival and growth of *L. donovani*.

ROS is known as a signaling molecule regulating autophagy in macrophages (Huang et al., 2011; Scherz-Shouval and Elazar, 2011). During infection, *Leishmania* parasites interfere with the oxidative stress mechanisms of macrophages for its persistence (Ghosh et al., 2003; Sansom et al., 2013). SUMOylation is also involved in maintaining ROS and NO homeostasis. Endogenous SUMO-1 is reported as a suppressor of ROS generation in neutrophils and smooth muscle cells (Akar and Feinstein, 2009; Pandey et al., 2011; Gupta et al., 2020). Taken this together, we evaluated the role of host SUMOylation in the regulation of generation levels of ROS and NO during *L. donovani* infection. We found that upon inhibition of any step, SUMOylation significantly elevated ROS generation in *L. donovani*-infected macrophages. Knockdown of *SUMO-1*, *UBA2*, and *UBC9* induced the production of NO upon the infection. The result suggests that host SUMOylation modulated the microbicidal mechanisms of macrophages in terms of autophagy and the levels of ROS generation and NO for the persistence of *Leishmania*. ROS acts as a key mediator to trigger inflammatory cytokine production for establishing and facilitating immune responses into either protection or non-protection toward the infection. As soon as the parasite invades, the host cell activates various cellular immune signaling pathways and inflammatory responses including Th1 and Th2 types to encounter the pathogen (Saha et al., 2007; Nylén and Gautam, 2010). The production level of IL-12 and IFN-γ downregulates while the level of IL-4 and IL-10 upregulates during the infection of *Leishmania* parasites (Murphy et al., 2001; Castellano et al., 2009). IL-10 blocks the activation of Th1 cells that suppress the production of IL-12 and IFN-γ to inhibit the parasite clearance mechanism (Bacellar et al., 2000). In splenic aspirate cells of VL patients, IFN-γ and TNF-α production upregulated by the neutralization of IL-10 decreases (Gautam et al., 2011) during parasite load. TNF-α leads to the stimulation of IFN-γ production (Singh et al., 2016) and has a potential role in granuloma formation, which facilitates the clearance of intracellular parasites (Titus et al., 1989; Murray, 2000). IL-12 is important for the production of IFN-γ from NK cells and T cells and induces NO generation and NOS2 expression, facilitating an antiparasitic effect (Liew and O’Donnell, 1993). SUMOylation is reported to regulate IFN responses by various molecular mechanisms (Hannoun et al., 2016; Crowl and Stetson, 2018). Many viral proteins manipulate the host SUMOylation pathway to establish infection by altering inflammatory responses (Kubota et al., 2008; Chang et al., 2009; Lowrey et al., 2017; Vidal et al., 2019). In light of these reports, we next investigated the inflammatory responses mediated by host SUMOylation during *L. donovani* infection. We found that the knockdown of genes of the host SUMOylation process significantly upregulates the production level of proinflammatory cytokines IL-12p40, IFN-γ, IL-32γ, and TNF-α, while the knockdown of deSUMOylating gene *SENP1* significantly downregulates the production levels of IL12p40 and TNFα. The finding suggests that host SUMOylation favors *Leishmania* infection by modulating pro-inflammatory immune responses. However, the knockdown of *SENP1* regulates the inflammatory responses and autophagy differently. Downregulation of *SENP1* leads to the reduction of IL-10 levels as well as upregulation of the expression of LAMP-1 and promotes the autophagosome–lysosome fusion in infected macrophages. Interestingly, knockdown of *SENP1* also reduces the level of IL-12p40 and TNF-α, but it does not modulate the generation levels of ROS and NO. These results suggest the collated effect of the interplay between pro-inflammatory and anti-inflammatory responses, which might lead to no changes in parasite load upon knockdown of *SENP1* in infected macrophages.

The most prominent alterations of the immune responses were observed upon the knockdown of *AOS-1*, *UBA2*, and *UBC9* in the *L. donovani*-infected macrophages. This could be because AOS1/UBA2 heterodimer and UBC9 are the sole E1-activating and E2-conjugating enzymes for SUMOylation, and inhibition of any of these enzymes can inhibit the overall SUMOylation process. Different isoforms of SUMO may have different or similar target protein preferences that may differentially regulate responses. A distinct and overlapping set of target proteins have been identified for SUMO-1 and SUMO-2, indicating their redundant and non-redundant cellular functions (Vertegaal et al., 2006). As observed in this study, the knockdown of *SUMO-1* and *SUMO-2/3* differentially regulates the process of autophagy initiation and autophagy maturation. Also, the knockdown of *SUMO-1* elevated the levels of ROS generation and NO more significantly than the knockdown of *SUMO-2/3* upon *L. donovani* infection, while the more significant upregulation of pro-inflammatory cytokines levels was observed upon the knockdown of *SUMO-2/3* compared to *SUMO-1*. Dissecting the SUMOylation pathway contributes to the understanding that in *Leishmania* infection, SUMOylation of various proteins *via* not only SUMO-1 but also SUMO-2 or SUMO-3 might play a role in differentially modulating various cellular immune responses of macrophages to facilitate the infection and growth of parasites.

**Figure 7:**
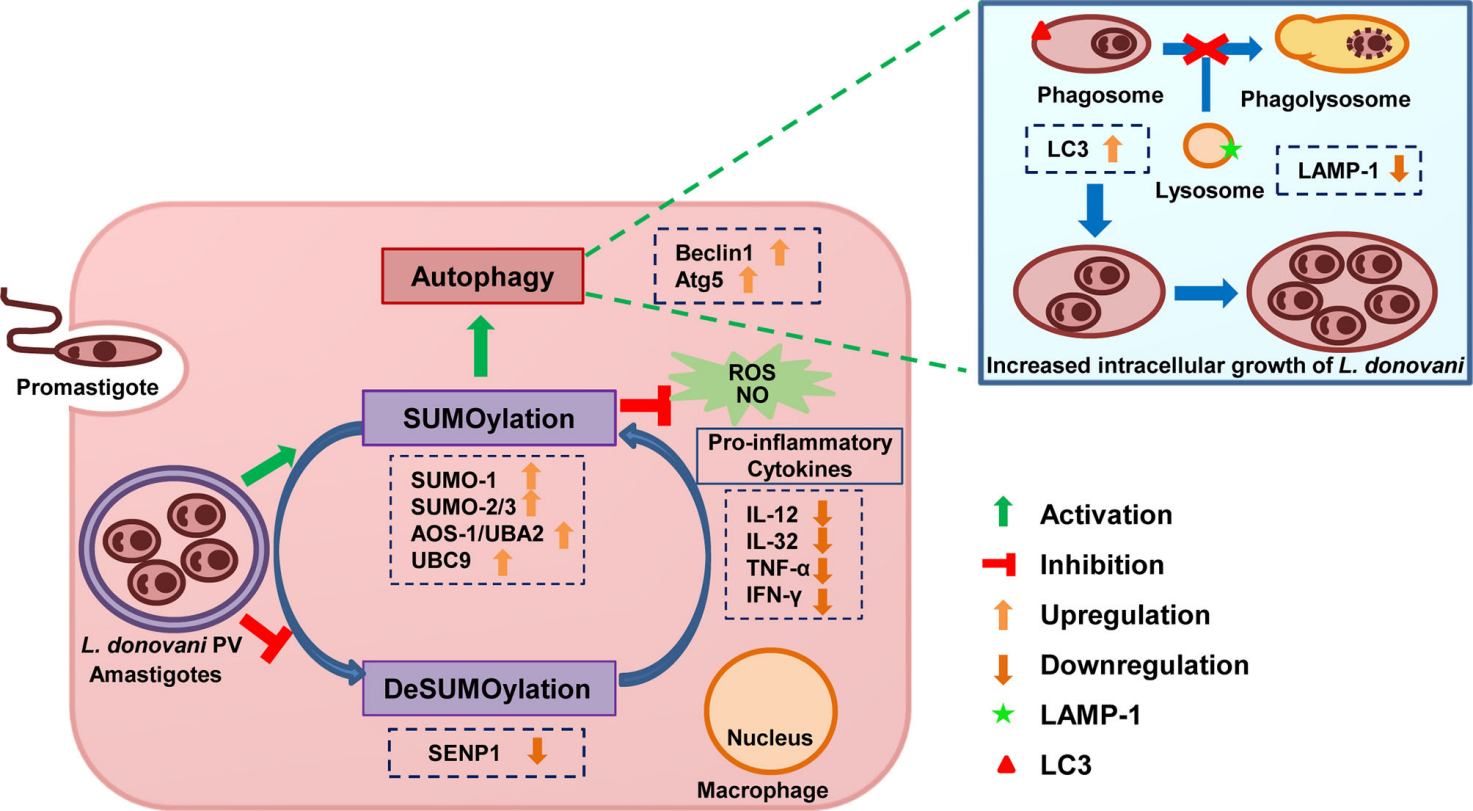
Schematic representation of *Leishmania donovani* survival and growth in the macrophages regulated by the host SUMOylation pathway. Host SUMOylation promotes autophagy initiation while suppressing autophagy maturation, ROS generation, and nitric oxide production as well the secretion of pro-inflammatory cytokines, thus favoring the parasite growth and survival. Targeting this pathway could be a potential target for developing novel drugs to restrict the parasite’s survival and growth. ROS, reactive oxygen species.

The results of this study demonstrate that *L. donovani* infection modulates the host SUMOylation process to facilitate its survival and growth in the macrophages by modulating host SUMOylation-mediated autophagy and the immune responses (**Figure 7**). This study also opens novel horizons for targeting the host SUMOylation pathway toward the development of therapeutic antileishmanial drugs.

## Data Availability Statement

The original contributions presented in the study are included in the article/**Supplementary Material**. Further inquiries can be directed to the corresponding authors.

## Author Contributions

JS, AR, and SS conceptualized the idea and designed the experiments. JS performed all the experiments. EM performed qRT-PCR and MTT assay. AC, PS, NS, SK, AKK, MM, MKM, and PJ helped in the experimental methodology. JS, AR, and SS critically analyzed the data. JS, AR, and SS wrote the manuscript. All authors agreed to the submitted version of the manuscript.

## Funding

This work has been funded by the Biotechnology Career Advancement & Re-orientation Program (BioCARe) by the Department of Biotechnology (BT/PR30534/BIC/101/1095/2018) sanctioned to JS. JS is a recipient of the BioCARe Women Scientist fellowship from DBT. This work is also supported by the Science and Engineering Research Board under the Department of Science and Technology (CRG/2019/002231, IPA/2020/000007) sanctioned to AR and SS. EM and AKK are supported by the CSIR-SRA fellowship. PS, MKM, and MM are supported by the ICMR-SRF fellowship. PJ is supported by the CSIR-JRF fellowship.

## Conflict of Interest

The authors declare that the research was conducted in the absence of any commercial or financial relationships that could be construed as a potential conflict of interest.

## Publisher’s Note

All claims expressed in this article are solely those of the authors and do not necessarily represent those of their affiliated organizations, or those of the publisher, the editors and the reviewers. Any product that may be evaluated in this article, or claim that may be made by its manufacturer, is not guaranteed or endorsed by the publisher.
