# Supplementary material for: Host SUMOylation Pathway Negatively Regulates Protective Immune Responses and Promotes Leishmania donovani Survival
Source: Front Cell Infect Microbiol. 2022 Jun 6;12:878136. doi: 10.3389/fcimb.2022.878136 (PMC9207379; doi:10.3389/fcimb.2022.878136)
Supplement: Supplementary file 1 [file DataSheet_1.doc]

**Host SUMOylation pathway negatively regulates protective immune responses and promotes *Leishmania donovani* survival**

**Jhalak Singhal^1*^, Evanka Madan^1^, Ayushi Chaurasiya^1^, Pallavi Srivastava^1^, Niharika Singh^1^, Shikha Kaushik^1^, Amandeep Kaur Kahlon^1^, Mukesh Kumar Maurya^1^, Manisha Marothia^1^, Prerna Joshi^1^, Anand Ranganathan^1*^, Shailja Singh^1*^**

^1^ Special Centre for Molecular Medicine, Jawaharlal Nehru University, New Delhi, India

*** Correspondence:** jhalakbiotech@gmail.com (J.S.); anand.icgeb@gmail.com (A.R.); shailja.jnu@gmail.com (S.S.)

**This file contains Supplementary Figure 1.**

**Supplementary Figure 1** THP-1 macrophages were transfected with specific siRNAs followed by with and without the stimulation of LPS (100ng/ml) for 24 hrs. RNA was isolated for gene expression analysis of inflammatory cytokines by quantitative real-time PCR (qRT-PCR). MOCK here represents the transfection with a control siRNA. Statistical significance was quantified using the unpaired t-test with Welch’s correction (**p*<0.05, ***p* < 0.01, and ****p* <0.001).
